# Transport‐associated pathway responses in ovine fetal membranes to changes in amniotic fluid dynamics

**DOI:** 10.14814/phy2.13455

**Published:** 2017-10-19

**Authors:** Cecilia Y. Cheung, Debra F. Anderson, Robert A. Brace

**Affiliations:** ^1^ Department of Obstetrics and Gynecology Oregon Health and Science University Portland Oregon; ^2^ Center for Developmental Health Oregon Health and Science University Portland Oregon

**Keywords:** Amniotic fluid volume, tubulin, vascular endothelial growth factor, vesicular transport

## Abstract

Current evidence suggests that amniotic fluid volume (AFV) is actively regulated by vesicular transport of amniotic fluid outward across the amnion and into the underlying fetal vasculature in the placenta. Our objective was to determine whether gene expression profiles of potential stimulators, inhibitors, and mediators of vesicular transport are altered in response to changes in intramembranous absorption (IMA) rate. Samples of ovine amnion and chorion were obtained from fetal sheep with normal, experimentally reduced or increased AFVs and IMA rates. Amnion and chorion levels of target mRNAs were determined by RT‐qPCR. In the amnion, caveolin‐1 and flotillin‐1 mRNA levels were unchanged during alterations in IMA rate. However, levels of both were significantly higher in amnion than in chorion. Tubulin‐*α* mRNA levels in the amnion but not in chorion were reduced when IMA rate decreased, and amnion levels correlated positively with IMA rate (*P* < 0.05). Dynamin‐2 mRNA levels were not altered by experimental conditions. Vascular endothelial growth factor (VEGF_164_ and VEGF_164_b) mRNA levels increased during both increases and decreases in IMA rate, whereas soluble Flt‐1 levels did not change. Neither HIF‐1*α* nor PBEF mRNA levels in the amnion were correlated with VEGF_164_ expression levels and were not related to IMA rate. Collectively, our findings suggest that changes in amnion microtubule expression may be important in the regulation of transcellular vesicular transport of amniotic fluid and thus modulate IMA rate. Further, our results are consistent with the concept that the amnion is the rate‐limiting layer for amniotic fluid transport.

## Introduction

The mechanisms that regulate amniotic fluid volume (AFV) remain not well understood even though disorders of AFV occur in 5–10% of human pregnancies and are associated with increased morbidity and mortality (Locatelli et al. 2004; Morris et al. 2014; Shrem et al. 2016; Khan and Donnelly 2017). Over the past 25 years, a growing body of evidence based on experimental animal studies has shown that the primary determinant of AFV is the rate of transport of amniotic fluid outward across the amnion into the underlying fetal vasculature that perfuses the fetal surface of the placenta (Gilbert and Brace 1989; Gilbert et al. 1997; Brace et al. 2004, 2014b; Adams et al. 2005; Brace and Cheung 2014). This process, termed intramembranous absorption (IMA), is dependent upon the transport characteristics of the amnion (Adams et al. 2005; Brace and Cheung 2011). A series of studies has shown that, although water and smaller solutes move passively in both directions across the intramembranous pathway, the majority of IMA is mediated by unidirectional vesicular transport of water and solutes (Adams et al. 2005; Gesteland et al. 2009; Brace et al. 2014a). This conclusion is supported by findings from electron and fluorescence microscopy studies of the amnion that observed pinocytotic vesicles with apparent transport characteristics (King 1993; Shandley et al. 1997; Shen et al. 2008). Further, when IMA rate was experimentally altered, only the vesicular component of IMA changed while the passive components were unaltered (Gesteland et al. 2009; Brace et al. 2014a).

Currently, little is known of the cellular and molecular processes that mediate vesicular transcytosis of amniotic fluid across the amnion. In a variety of mammalian cells including endothelial, epithelial and cancer cells, transport vesicles including caveolae, lipid rafts and clathrin‐coated vesicles have been described (Li et al. 2013; Bitsikas et al. 2014; Kirchhausen et al. 2014). Our recent study, using fluorescence super‐resolution microscopy, has identified caveolar and clathrin‐coated vesicles in human amnion that exhibited uptake and transport behavior consistent with intramembranous absorption of amniotic fluid (Sharshiner et al. 2017). However, the mechanisms that mediate this vesicular uptake and transport are unclear. The dependence of transport rate on abundance of vesicles, rate of internalization of vesicles from the plasma membrane, or changes in microfilamental structures along which vesicles are transported has not been explored. Further, although experimental studies demonstrated the presence of powerful stimulator(s) and inhibitor(s) of IMA in amniotic fluid (Anderson et al. 2013; Brace et al. 2014b), the identity of these regulators has yet to be determined and the associated signaling mediators have not been identified (Cheung et al. 2014). Finally, even though the amnion has been proposed as the rate‐limiting barrier, the involvement of the chorion in mediating changes in IMA has not been investigated and it is not clear whether changes in molecular profiles in the chorion parallel those in the amnion.

In order to address these gaps in understanding, this study conducted in chronically catheterized ovine fetuses was designed with three aims. Our first aim was to gain insight into the trans‐amnion transport processes. To do this, we examined several potential factors that may be involved in regulating vesicle number and transport in the amnion. For exploring the types of transport vesicles, we selected caveolin‐1 as a marker for caveolar vesicles (Gross et al. 2017), and flotillin‐1, an integral membrane protein associated with planar lipid rafts (Salzer and Prohaska 2001). These principal components are known to be widely expressed in many cell types to regulate vesicle transport functions (Head et al. 1838; Chen et al. 2002). To study vesicle internalization and release, we selected the vesicle fission effector dynamin‐2 that mediates vesicle separation from the plasma membrane (Henley et al. 1998). For investigating transcellular vesicle movement specifically via the microtubule pathway, we focused on tubulin‐*α*, an essential constituent of microtubules (Heald and Nogales 2002).

The second aim of this study was to explore whether vascular endothelial growth factor (VEGF) participated in the cell signaling pathways that regulated IMA rate and determine whether known regulators of VEGF may function as the purported stimulator(s) and inhibitor(s) of IMA. Our early studies in fetal sheep demonstrated that VEGF mRNA levels in the amnion were elevated whenever IMA rate was experimentally increased (Cheung 2004). In other studies of endothelial and epithelial cells, exogenous VEGF has been shown to increase permeability by inducing caveolar transport (Feng et al. 1999; Chen et al. 2002; Harvey et al. 2016). The proposed mechanism was a VEGF‐ mediated caveolar endocytosis. Further, we previously reported that VEGF activated caveolin‐1 phosphorylation in ovine amnion cells (Cheung et al. 2010). However, caveolin‐1 is known to negatively regulate nitric oxide synthase activity (Liu et al. 2017) and caveolin‐1 mRNA levels were reduced in fetal sheep when IMA was elevated during fetal hypoxia (Cheung and Brace 2008). These opposing actions of caveolin‐1 and its interactions with VEGF raised the need to explore the relationships among VEGF, caveolin‐1 and IMA rate in the amnion. In order to determine whether known activators of VEGF would modulate IMA rate through VEGF expression, we selected two factors: hypoxia inducible factor‐1*α* (HIF‐1*α*) which activates VEGF transcription (Forsythe et al. 1996), and pre‐*β* cell colony‐enhancing factor (PBEF) which up‐regulates VEGF gene expression and increases VEGF receptor activities in amnion cells (Astern et al. 2013). In addition, we tested two potential inhibitors of VEGF activities: soluble fms‐like tyrosine kinase‐1 (sFlt‐1, sVEGF‐R1) (Kendall and Thomas 1993) which binds VEGF thus reduces VEGF bioavailability, and VEGF_164_b, a VEGF_164_ isoform formed by alternate splicing of the VEGF gene, that antagonizes the VEGF receptor‐mediated downstream signaling pathways (Nowak et al. 2008; Ngo et al. 2014).

The third aim of this study was to determine whether experimentally induced changes in gene expression profile of vesicular transport components in the amnion would also occur in the chorion. Although the amnion has been recognized as the rate‐limiting barrier for IMA, the chorion is in intimate contact with the outer surface of the amnion and is vascularized by fetal blood vessels in the ovine model (Brace et al. 1992). As such, the chorion could potentially participate in the regulation of intramembranous transport of amniotic fluid.

In order to address the above 3 aims, we determined the gene expression pattern of the selected mediators of IMA in the amnion and chorion of ovine fetuses subjected to experimentally induced modifications in IMA rate and AFV. The hypotheses tested were that experimentally produced changes in IMA rate would be associated with (1) alterations in expression of vesicle‐associated mediators and microtubule components that facilitate transcellular vesicular transport; (2) changes in VEGF mRNA levels that are consistent with the induced changes in IMA rate and AFV, and that the VEGF regulators tested could function as the proposed stimulator(s) and inhibitor(s) of IMA; and (3) changes in gene expression profiles that are specific to the amnion but not the chorion, supporting the concept that amnion is the rate‐limiting layer for amniotic fluid transport.

## Materials and Methods

### Ovine tissues

We utilized amnion and chorion tissues of late gestation ovine fetuses obtained from recent studies in chronically catheterized fetal sheep. The experimental protocols were described elsewhere (Cheung et al. 2016). Tissues were collected 2 days after initiating one of four different experimental conditions: control, urine drainage without fluid replacement, urine drainage with isovolumic fluid replacement (lactated Ringer's solution), and continuous intra‐amniotic fluid infusion (2 L/day of lactated Ringer's solution). AFV was measured at the beginning and end of the 2‐day experimental period and IMA rate, measured as a mean over the 2 days, was determined from the change in AFV and the time integrated amniotic inflows and outflows (Robertson et al. 2009; Anderson et al. 2013). The experimental design was based on our observations that, compared to control conditions, urine drainage reduces IMA and AFV, urine replacement reduces IMA and increases AFV, and intra‐amniotic fluid infusion increases both IMA and AFV (Anderson et al. 2013; Brace et al. 2014a).

### Quantitative reverse transcription‐polymerase chain reaction (RT‐qPCR)

Total RNA was extracted from amnion and chorion tissues using an RNeasy Kit (Qiagen, Inc., Valencia, CA). The relative quantities of target mRNA in the tissues were determined by RT‐qPCR for caveolin‐1, flotillin‐1, dynamin‐2, tubulin‐*α*, VEGF_164_, and VEGF_164_b, sFlt‐1, PBEF and HIF‐1*α*. The methodologies were similar to those recently described (Cheung et al. 2016). Single strand cDNA synthesis from total RNA (2 *μ*g) was carried out using MultiScribe reverse transcriptase and random primers in the presence of RNase inhibitor (Applied Biosystems, Life Technologies, Foster City, CA). Sample cDNA (25 ng/sample) was amplified with ovine‐specific primers and probes custom designed using Primer Express^®^ Software v3.0 (Applied Biosystems, Thermo Fisher Scientific) (Table 1). The amplified sequences were validated by sequencing and alignment to the consensus ovine sequences. For the amplification reaction, *TaqMan* Gene Expression Assays (Applied Biosystems, Thermo Fisher Scientific) were used in a ViiA7 Real‐Time PCR System (Applied Biosystems). Two endogenous references, 18S ribosomal RNA and ovine RPLP0 mRNA, were used as house‐keeping genes. For each endogenous reference and target gene, a standard curve was incorporated in order to yield accurate quantitative values needed for comparative analysis. The mean of triplicate *C*_T_ values interpolated from the respective standard curves were corrected for amplification efficiency. The Δ*C*_T_ for each of the target mRNAs was calculated by subtracting the geometric mean *C*_T_ of the 2 endogenous reference genes. The fold change (2^−∆∆^C_T_) of target gene relative to the respective ∆*C*_T_ in amnion under control condition (the latter is used as the calibrator) was determined by the Comparative *C*_T_ method (Schmittgen and Livak 2008), where ∆*C*_T_ denotes the target *C*_T_ value referenced to the house‐keeping genes and ∆∆*C*_T_ the difference from ∆*C*_T_ in control amnion.

**Table 1:** Custom‐designed ovine specific probes and primers used for real‐time quantitative RT‐PCR determinations of target mRNA levels in ovine fetal tissues

Gene	Primer/probe	Nucleotide sequence	Amplicon size
VEGF_164_	Forward	GGCGAGGCAGCTTGAGTTAA	75
	Reverse	CACCGCCTCGGCTTGTC	
	Probe	6FAMCGAACGTACTTGCAGATGMGBNFQ	
VEGF_164_b	Forward	AACACAGACTCGCGTTGCAA	63
	Reverse	GGTGAGACGTCTGCAAGTACGTT	
	Probe	6FAMCGAGGCAGCTTGAGMGBNFQ	
sFlt‐1	Forward	TGCCGAGCTAGGAACATATACACA	103
	Reverse	AGATCCGAGAGAAAACAGCCTTT	
	Probe	6FAMAAGAAATCCTCCAGAAGAAMGBNFQ	
Caveolin‐1	Forward	TGTGATTGCAGAACCAGAAGGA	61
	Reverse	GGTGAAGCTGGCCTTCCA	
	Probe	6FAMCACACAGTTTCGATGGCMGBNFQ	
Flotillin‐1	Forward	CACAGAGGGACTACGAGCTGAA	58
	Reverse	CGGCGCGTGTTGACTTC	
	Probe	6FAMAAGGCCGCATACGACMGBNFQ	
Dynamin‐2	Forward	CGGCTGAGAGGAAGTTTTTCC	61
	Reverse	CCCATGCGGTCCGCTAT	
	Probe	6FAMACCCAGCCTACCGGCMGBNFQ	
Tubulin‐*α*	Forward	ACGTGGTTCCCAAAGATGTCA	66
	Reverse	GCTGCGCTTGGTCTTGATG	
	Probe	6FAMTGCTGCCATTGCCMGBNFQ	
HIf‐1*α*	Forward	CCATGCCCCAGATTCAAGAT	59
	Reverse	ACTTTGTCTGGTGCTTCCATCA	
	Probe	6FAMAGCCAGCTAGTCCTTMGBNFQ	
PBEF	Forward	TGATCCCAACAAAAGGTCCAA	63
	Reverse	AAATTTCCTGCTGGTGTCCTATG	
	Probe	6FAMAAGGGCCGATTATCMGBNFQ	

### Statistics and data presentation

Data are presented as means ± SE. Paired and unpaired t‐tests as well as parametric one and two factor ANOVAs were used for statistical comparisons. For the two factor ANOVAs, the interaction term indicates whether changes in the two tissues differed with experimental condition. Post hoc testing utilized Fisher's least significant difference if the null hypothesis was rejected. Bivariate (Brace 1977) and multivariate least squares regression were used to explore relationships between and among mRNA levels and IMA rate. Pearson's correlation coefficient (*r*) was used to determine the closeness of fit to a linear relationship of the data. Logarithmic transformation was used to normalize variances prior to statistical testing as needed. The relative amounts of mRNA were analyzed after logarithmic transformation; this method yielded identical statistical results and graphs as analyses using ΔΔ*C*_T_ values or Δ*C*_T_ values with the exception that the scale on the vertical axis changed. A probability value *P* ≤ 0.05 was considered significant. Regressions and correlations that were not statistically significant are not shown.

## Results

Experimental values of IMA rate and AFV as well as their ranges are given in Table 2. Urine drainage reduced IMA rate and AFV below control levels while intra‐amniotic fluid infusion increased both above control (*P* < 0.05, ANOVA). The decrease in mean IMA rate and increase in AFV following urine replacement with lactated Ringer's solution were not statistically significant.

**Table 2:** Intramembranous absorption rates and amniotic fluid volumes in late gestation ovine fetuses under four experimental conditions (*n* = 4 per group)^a^

Experimental conditions	Intramembranous absorption rate (mL/day)		Amniotic fluid volume (mL)	
	Mean ± SE	Range	Mean ± SE	Range
Control	667 ± 159	395–1075	1110 ± 125	865–1449
Urine drainage	101 ± 120*	−190–394	300 ± 77*	128–437
Urine replacement	515 ± 29	453–567	1449 ± 220	864–1862
Intra‐amniotic infusion^b^	1366 ± 274*	813–1945	3351 ± 365*	2351–4101

*P <* *0.05 compared to respective control.

a Data from Cheung et al. (2016).

b Lactated Ringer's solution infused at 2 L/day (1.39 mL/min).

**Figure 1:**
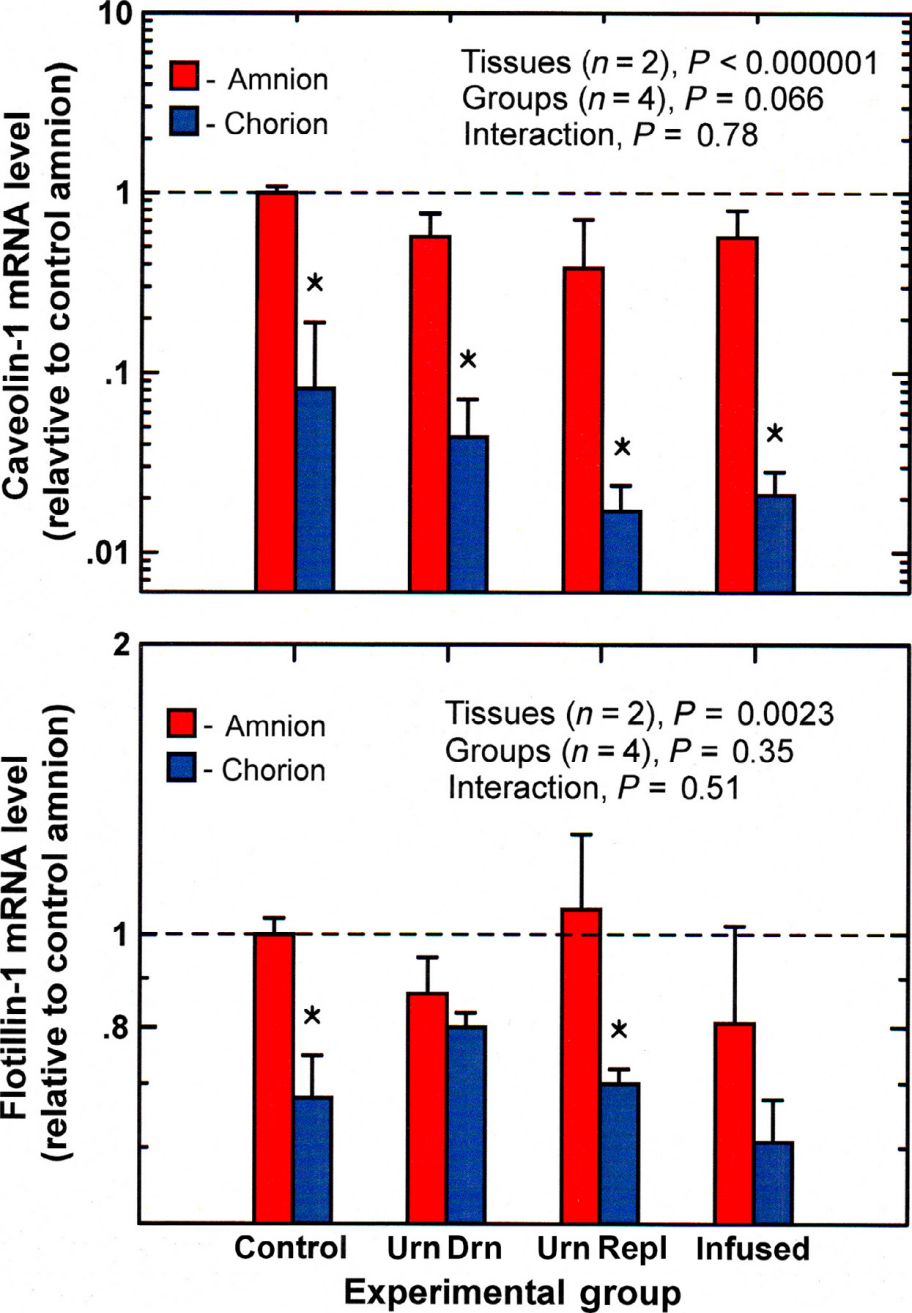
Caveolin‐1 and flotillin‐1 mRNA levels (mean ± SE,* n* = 4) in ovine amnion and chorion under control and experimental conditions. Data in this and subsequent figures are normalized to mean mRNA levels in control amnion (horizontal dashed line) urn drain, continuous drainage of fetal urine without fluid replacement; urn repl, urine drainage with isovolumic fluid replacement; infused, continuous infusion of lactated Ringer's solution (2 L/day) into the amniotic fluid. *P < 0.05, chorion level compared to amnion level for the same experimental group.

Caveolin‐1 mRNA levels in the amnion did not change significantly with experimental condition (Figure 1) and were unrelated to IMA rate for all four experimental groups combined (*n* = 16, *P* = 0.99). In the chorion, caveolin‐1 mRNA levels were not correlated with IMA rate (*P* = 0.61). Caveolin‐1 mRNA levels in the amnion were much higher than those in the chorion for all experimental groups (*P* < 0.0001, Fig. 1).

Neither in the amnion nor in chorion did flotillin‐1 mRNA levels change significantly from control during experimental conditions (Fig. 1). Flotillin‐1 mRNA levels in the amnion were higher than in the chorion (*P* = 0.0023) but were not related to IMA rate (*P* = 0.42). In contrast, chorion flotillin‐1 mRNA levels were negatively correlated with IMA rate (*n* = 16, *r* = −0.59, *P* < 0.05).

**Figure 2:**
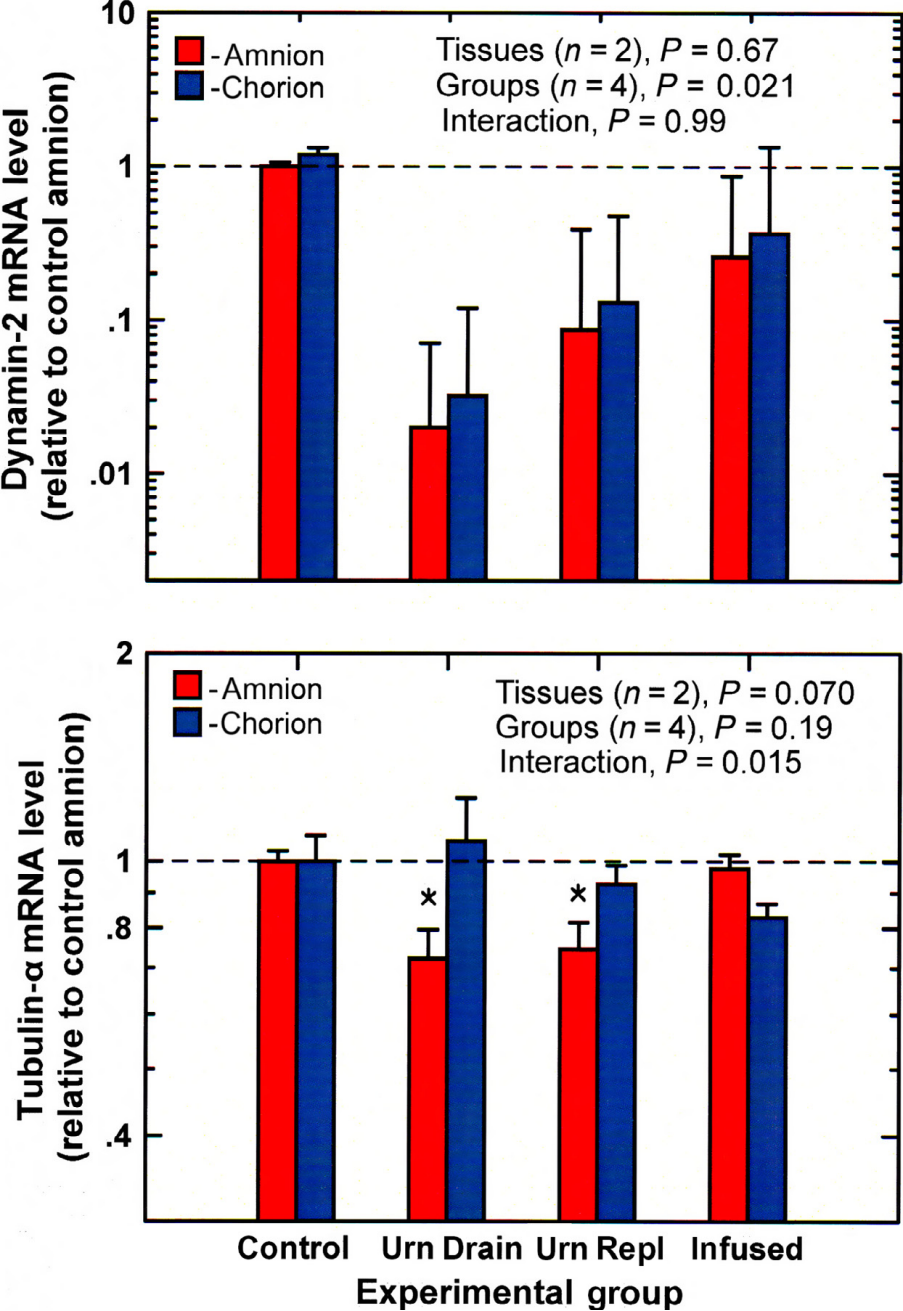
Dynamin‐2 and tubulin‐*α* mRNA levels (mean ± SE,* n* = 4) in ovine amnion and chorion under control and experimental conditions urn drain, continuous drainage of fetal urine without fluid replacement; urn repl, urine drainage with isovolumic fluid replacement; infused, continuous infusion of lactated Ringer's solution (2 L/day) into the amniotic fluid. *P < 0.05 compared to control amnion.

In the amnion and chorion, dynamin‐2 mRNA levels were unchanged under experimental conditions compared to control (Fig. 2). Even though the ANOVA suggested significant differences among groups, post hoc testing found no significance between individual groups. Dynamin‐2 levels in both amnion (*P* = 0.73) and chorion (*P* = 0.46) were not correlated with IMA rate.

**Figure 3:**
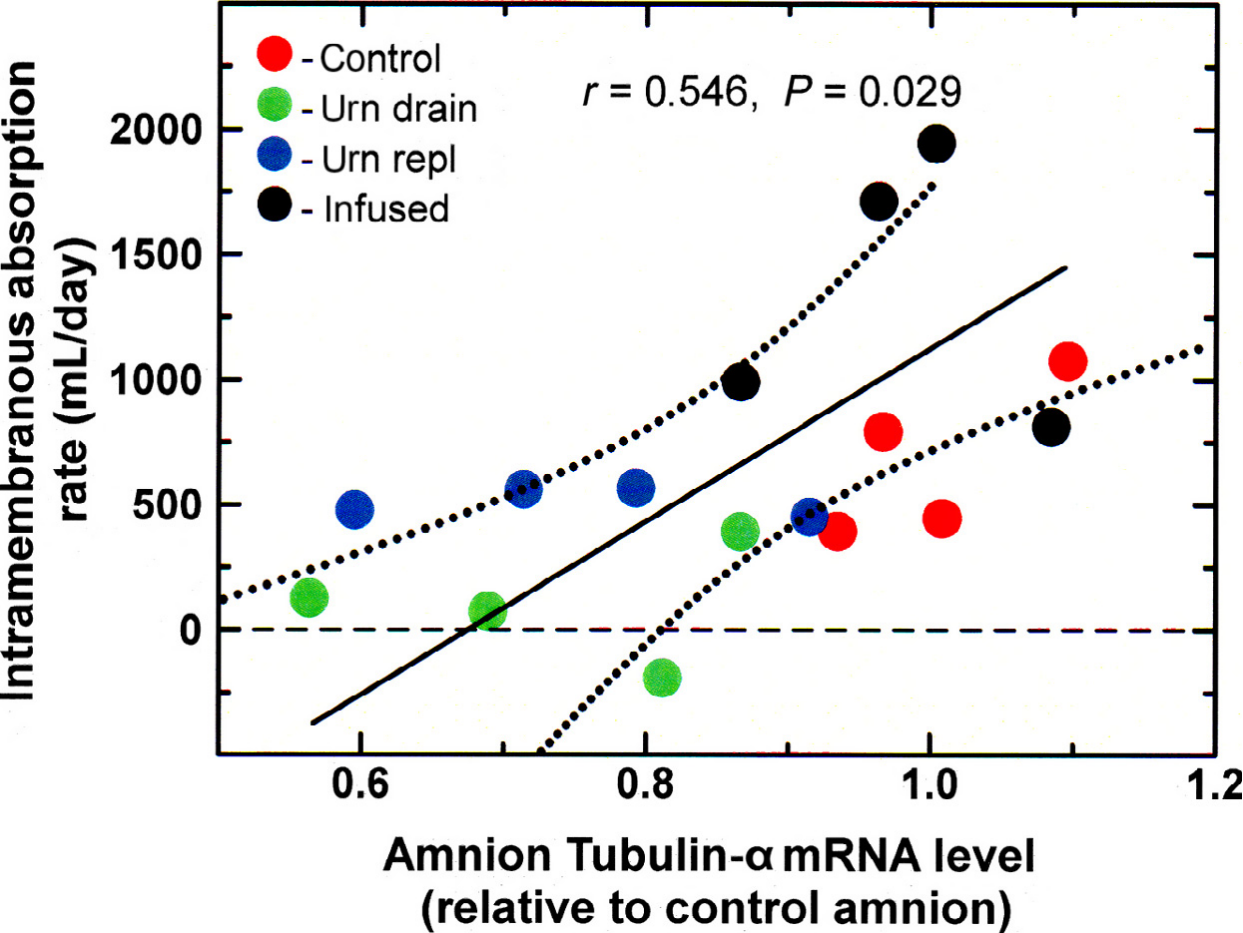
Regression relationship between amnion tubulin‐*α* mRNA levels and IMA rate. Solid line, regression line; dotted lines, 95% confidence interval about the regression line.

Tubulin‐*α* mRNA levels in the amnion were significantly reduced during urine drainage and urine replacement (ANOVA, *P* < 0.05) but did not change with intra‐amniotic fluid infusion (Fig. 2). In the chorion, tubulin‐*α* levels were not significantly altered by experimental conditions. Tubulin‐*α* mRNA levels in the amnion were positively correlated with IMA rate (*n* = 16, *r* = 0.546, *P* = 0.029, Fig. 3), while levels in the chorion were not (*P* = 0.17).

**Figure 4:**
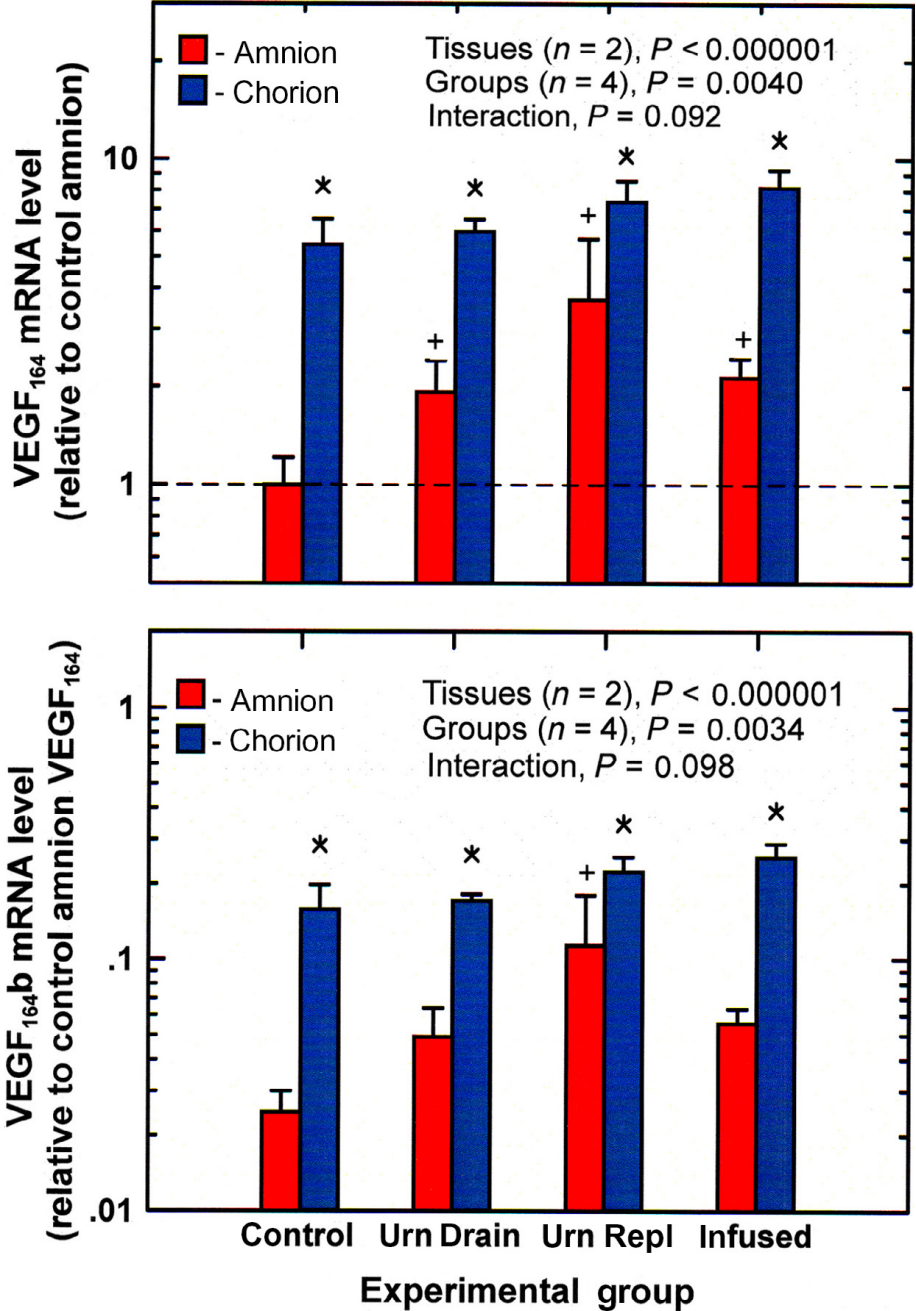
VEGF_164_ and VEGF_164_b mRNA levels (mean ± SE,* n* = 4) in ovine amnion and chorion under control and experimental conditions urn drain, continuous drainage of fetal urine without fluid replacement; urn repl, urine drainage with isovolumic fluid replacement; infused, continuous infusion of lactated Ringer's solution (2 L/day) into the amniotic fluid. ^+^ *P* < 0.05 compared to control amnion; *P < 0.05 chorion level compared to amnion level for the same experimental group.

The VEGF_164_ mRNA levels in the amnion were elevated during intra‐amniotic fluid infusion compared to control (ANOVA, *P* < 0.05). However, levels were also elevated during urine drainage and urine replacement (Fig. 4). In the chorion, VEGF_164_ mRNA levels were significantly higher than in the amnion under all conditions, averaging 4.00 ± 0.59 times (*P* < 0.0001). However, chorionic levels did not vary with experimental condition. Neither amnion (*P* = 0.87) nor chorion (*P* = 0.23) VEGF_164_ levels correlated with IMA rate. The relationship between VEGF_164_ and caveolin‐1 was determined and found to correlate negatively in the amnion (*r* = −0.52, *P* = <0.05) but not in the chorion (*P* = 0.08).

The changes in VEGF_164_b mRNA levels essentially paralleled those of VEGF_164_ under all experimental conditions (Fig. 4). However, VEGF_164_b levels were significantly lower. The VEGF_164_b mRNA abundance averaged 2.60 ± 0.05% and 2.98 ± 0.06% (*n* = 16, *P* < 0.0001) of VEGF_164_ mRNA levels in the amnion and chorion, respectively. In both tissues, there were no correlations between VEGF_164_b mRNA levels and IMA rate (*P* = 0.85 for amnion, *P* = 0.17 for chorion). For the combined amnion and chorion values (*n* = 32), VEGF_164_b levels were highly correlated with that of VEGF_164_ (*r* = 0.993, *P* < 0.0001).

**Figure 5:**
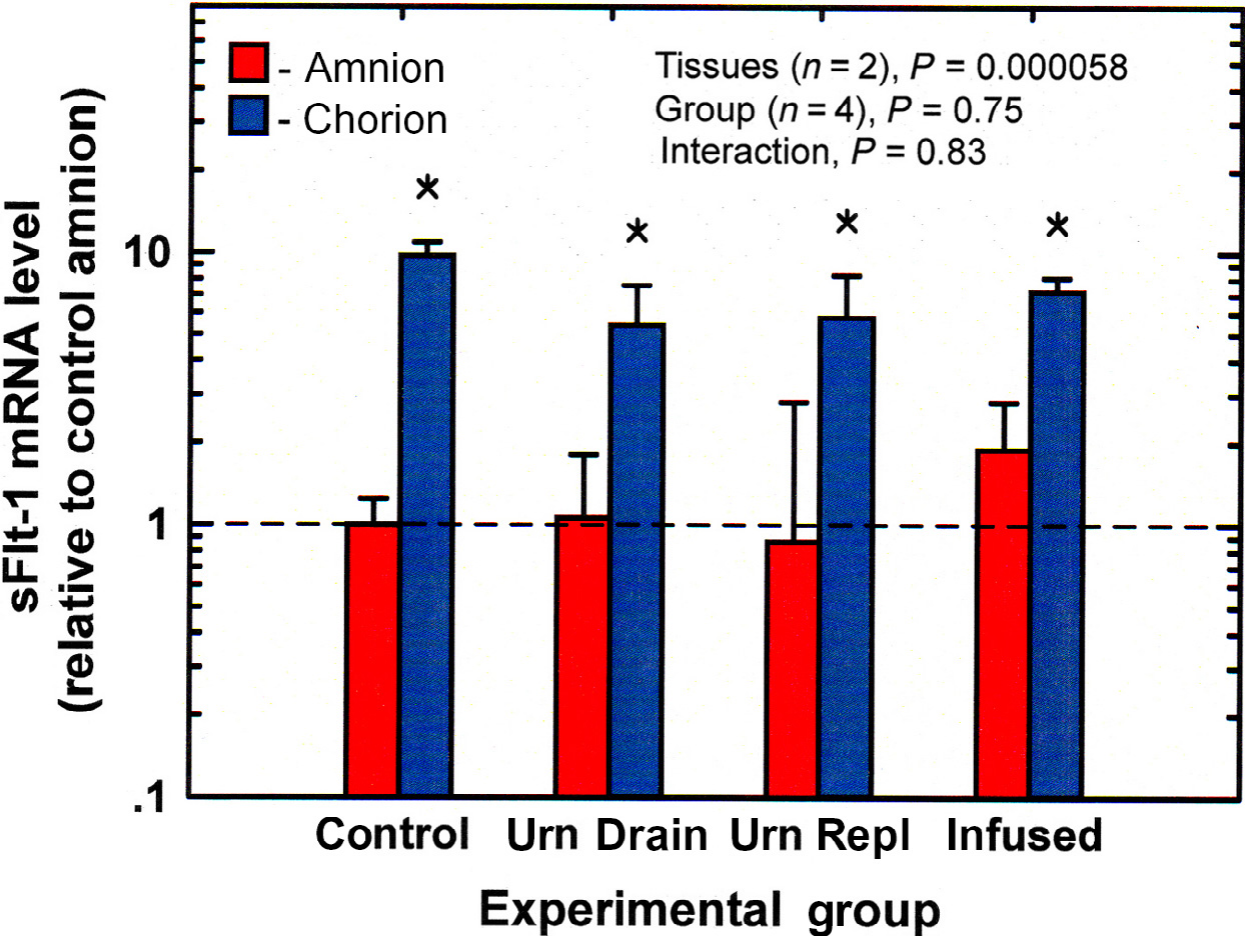
Soluble Flt‐1 mRNA levels (mean ± SE,* n* = 4) in ovine amnion and chorion under control and experimental conditions urn drain, continuous drainage of fetal urine without fluid replacement; urn repl, urine drainage with isovolumic fluid replacement; infused, continuous infusion of lactated Ringer's solution (2 L/day) into the amniotic fluid. *P < 0.05 chorion compared to amnion for the same experimental group.

Amnion mRNA levels of sFlt‐1 were higher than those in the chorion (*P* < 0.0001, Fig. 5). The levels in both tissues did not change with experimental conditions and were not correlated with IMA rate (*P* = 0.58 for amnion, *P* = 0.74 for chorion).

**Figure 6:**
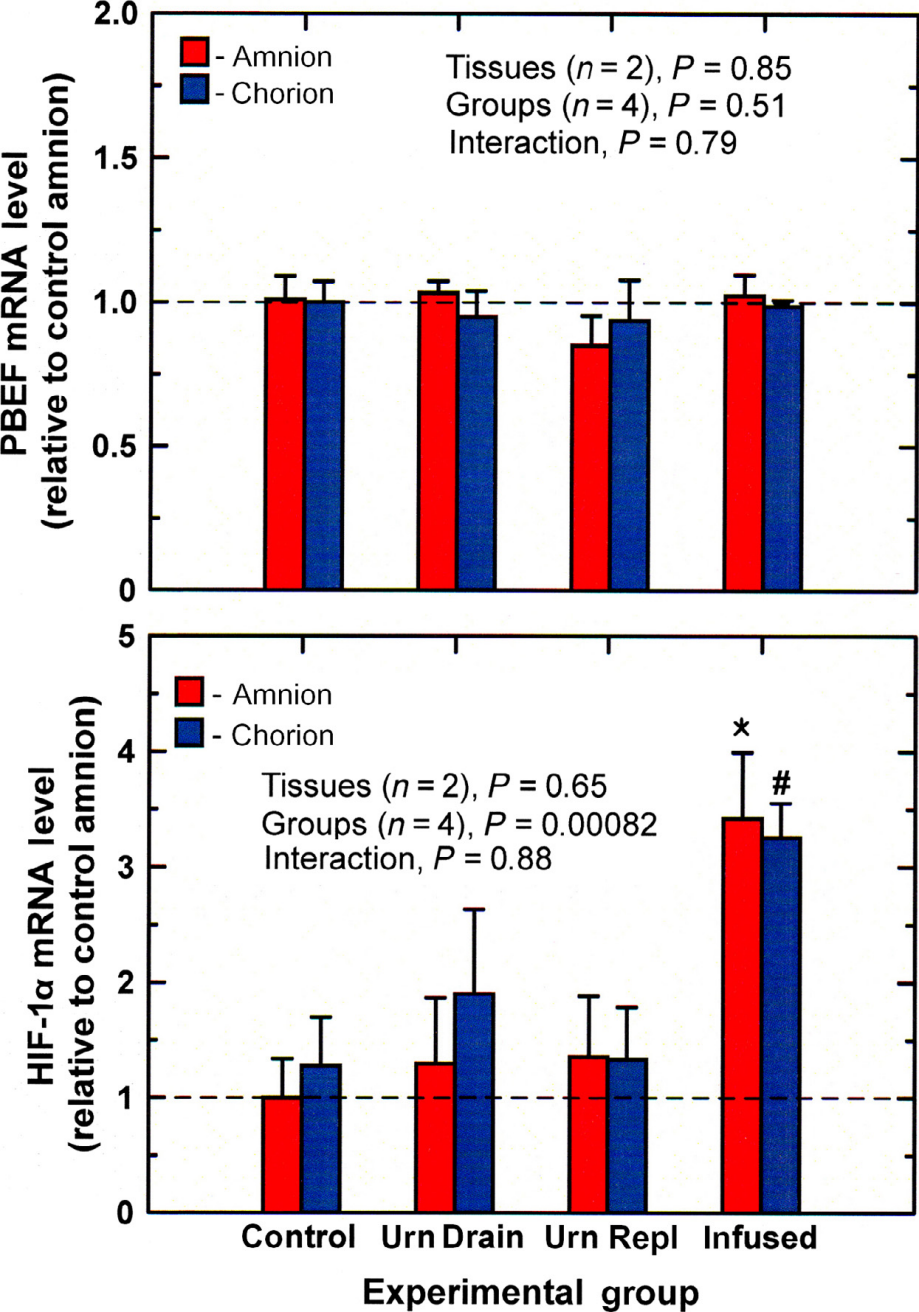
PBEF and HIF‐1*α* mRNA levels (mean ± SE,* n* = 4) in ovine amnion and chorion under control and experimental conditions urn drain, continuous drainage of fetal urine without fluid replacement; urn repl, urine drainage with isovolumic fluid replacement; infused, continuous infusion of lactated Ringer's solution (2 L/day) into the amniotic fluid. *P < 0.001 compared to control amnion; ^#^ *P* < 0.001, compared to control chorion.

PBEF mRNA levels were not altered with experimental conditions in either amnion or chorion (Fig. 6). There was no significant correlation between PBEF and VEGF_164_ mRNA levels in the amnion (*P* = 0.48) or chorion (*P* = 0.95); and neither amnion (*P* = 0.39) nor chorion (*P* = 0.63) PBEF mRNA levels correlated with IMA rate. Hypoxia inducible factor ‐1*α* mRNA levels in the amnion and chorion under the four experimental conditions are shown in Figure 6. HIF‐1*α* mRNA levels were elevated in both tissues during intra‐amniotic infusion (ANOVA, *P* < 0.001). Neither amnion nor chorion HIF‐1*α* mRNA levels correlated with VEGF_164_ (*P* = 0.47 for amnion, *P* = 0.13 for chorion), but amnion HIF‐1*α* levels positively correlated with IMA rate (*n* = 16, *r* = 0.57, *P* < 0.05) while chorion levels did not (*P* = 0.23).

When multivariate regression analysis of mRNA quantities for all target genes in the amnion and the chorion were performed, IMA rate was positively correlated with tubulin‐*α* in the amnion and negatively with flotillin‐1 in the chorion (*R* = 0.72, *P* < 0.01).

## Discussion

Previous studies have provided substantial evidence supporting the concept that IMA is mediated primarily by unidirectional vesicular transport of amniotic fluid outward across the amnion (Brace et al. 2014a). In this study, we explored the cellular and molecular mechanisms that potentially underlie changes in vesicular transport rate that would lead to large changes in IMA as induced experimentally (Gesteland et al. 2009; Brace and Cheung 2014).

In the amnion, the absence of change in expression levels of caveolin‐1, a structural component of caveolae (Galbiati et al. 1998), and flotillin‐1, an integral membrane protein of lipid rafts (Salzer and Prohaska 2001), together with the lack of correlation with IMA rate suggests that vesicle number might not be altered by experimental conditions. This implies that vesicular transport may not be dependent on the number of caveolae or lipid rafts in the amnion. In the chorion, the negative correlation between IMA rate and flotillin‐1 expression may suggest a response to changes in transport. However, our previous studies (Adams et al. 2005) indicated that transfer across the chorion was rapid rather than rate‐limiting under all experimental conditions and thus most likely not a site of regulation.

It is important to note that both caveolin‐1 and flotillin‐1 expression levels in the amnion were much higher than in the chorion suggesting that vesicles may be more abundant in the amnion consistent with a unique transport function of this tissue.

During endocytosis, dynamin‐2 participates in the cleavage and release of membrane invaginations to form intracellular vesicles (Henley et al. 1998). Although amnion dynamin‐2 mRNA levels tended to be lowered in all experimental groups compared to control, the lack of correlation with IMA rate does not support a major role for dynamin‐2 in mediating the experimentally induced changes in IMA.

Tubulins are structural components of microtubules that facilitate intracellular vesicle trafficking (Heald and Nogales 2002). The present finding that amnion tubulin‐*α* mRNA levels decreased when IMA rate was reduced during urine drainage and urine replacement suggests that a decline in structural integrity/number of microtubules may contribute to the reduction in transport rate under these conditions. This premise is supported by the positive correlation between amnion tubulin‐*α* mRNA levels and IMA rate by both bivariate and multivariate regression analyses. However, the lack of increase in tubulin‐*α* mRNA during intra‐amniotic infusion when IMA rate was elevated suggests additional factors may be involved. Importantly, since tubulin‐*α* level in the chorion was not affected by experimental conditions nor related to IMA rate, it appears that the response observed in the amnion was specific to the amnion, further supporting the concept that tubulin may play an important role in mediating intramembranous transport across the amnion and ultimately regulate AFV.

As anticipated (Cheung 2004), VEGF_164_ mRNA levels in the amnion were elevated as IMA rate increased. However, rather than being reduced during reductions in IMA rate as expected, VEGF_164_ mRNA levels were significantly increased. This suggests that factors other than VEGF may be involved in regulating IMA rate under experimental conditions. The mRNA levels of VEGF_164_b were detectable in ovine amnion; however, the levels were low representing only 3% of VEGF_164_ mRNA levels. Additionally, the changes in amnion VEGF_164_b mRNA levels paralleled those of VEGF_164_ in response to experimental conditions instead of being inversed and were not related to IMA rate. This indicates not only that the changes in expression levels of the 2 isoforms could be the consequence of a common mechanism rather than a negative relationship but also that VEGF_164_b would not be participating in regulation of IMA rate.

Both VEGF_164_ and VEGF_164_b were more abundantly expressed in the chorion than in the amnion consistent with previous findings (Cheung et al. 1995). Since chorion levels did not change with experimental conditions and were not related to IMA rate, it appears that chorion VEGF may not be participating in modulating amniotic fluid transport. In this study, we did not detect a decrease in sFlt‐1 mRNA levels in the amnion when IMA rate was elevated, suggesting that sFlt‐1 was not interacting with VEGF to modify intramembranous transport as anticipated. This was consistent with the lack of relationship between sFlt‐1 and IMA rate.

Previous investigators have shown that application of VEGF to retinal endothelial cells increased permeability by mediating caveolar transport (Feng et al. 1999). We postulated that VEGF would similarly mobilize caveolae in amnion epithelial cells by up‐regulating caveolin‐1. However, in this study, caveolin‐1 mRNA level in the amnion was negatively correlated with VEGF_164_ mRNA. This finding does not support the notion that induction of VEGF would activate caveolin‐1 to enhance caveolar transcytosis.

We explored the effects of modifications in IMA rate on two known factors of VEGF activation: HIF‐1*α* (Forsythe et al. 1996) and PBEF (Astern et al. 2013). Our results did not detect significant changes in PBEF mRNA levels as IMA rate varied by more than an order of magnitude. On the other hand, HIF‐1*α* mRNA levels increased during elevated IMA rates in the absence of hypoxia. However, the lack of correlation between amnion HIF‐1*α* or PBEF mRNA levels with VEGF_164_ levels imply that these factors were minimally involved in regulating the expression of VEGF under each of the four experimental conditions.

## Conclusions

Overall our findings provided new understanding of the transport components that are involved in the regulation of vesicular fluid movement across the amnion. First, the lack of change in caveolin‐1, flotillin‐1, or dynamin‐2 does not support the notion that alterations in the number of transport vesicles or the rate of their release from the plasma membrane was the underlying cause of the changes in IMA rate induced experimentally. Second, VEGF levels were not correlated with IMA rate, suggesting additional regulatory factors participate in the modulation of trans‐amnion vesicular transport. Third, the lack of a positive relationship between VEGF and its activators HIF‐1*α* and PBEF or a negative correlation with the antagonists VEGF_164_b and sFlt‐1 suggests that these VEGF agonists and antagonists would not be the unidentified stimulator(s) and inhibitor(s) of IMA found in the amniotic fluid. Fourth, the expression levels of caveolin‐1 and flotillin‐1 were significantly higher in the amnion than chorion, suggesting that transport vesicles may be more abundant in the amnion than chorion. Finally, an important finding in this study was the significant positive relationship between amnion tubulin‐*α* and IMA rate, and the down‐regulation of tubulin‐*α* when IMA rate was reduced. These responses were unique to the amnion and not detected in the chorion consistent with the concept that amnion is the rate‐limiting layer for amniotic fluid transport. Therefore, we conclude that intramembranous transport rate across the amnion may be regulated by the number, integrity and/or activity of intracellular microtubules.

## Conflict Of Interest

There are no conflicts of interest for any of the authors. There is no information for disclosure.
